# Is the Blood Pressure Paradox Observed in All Heart Failure Patients?

**DOI:** 10.1155/2013/350289

**Published:** 2013-11-25

**Authors:** F. M. Cunha, P. Lourenço, M. Couto, P. Tavares, S. Silva, J. T. Guimarães, P. Bettencourt

**Affiliations:** ^1^Serviço de Medicina Interna, Centro Hospitalar de São João, Alameda Professor Hernâni Monteiro, 4202-451 Porto, Portugal; ^2^Unidade I&D Cardiovascular do Porto, Faculdade de Medicina da Universidade do Porto, 4202-451 Porto, Portugal; ^3^Serviço de Patologia Clínica, Centro Hospitalar São João, Departamento de Bioquímica da Faculdade de Medicina da Universidade do Porto, 4202-451 Porto, Portugal

## Abstract

*Background.* Heart failure (HF) patients with higher systolic blood pressure (SBP) survive longer. Diabetes mellitus (DM) is a frequent comorbidity in HF. We evaluated the prognostic significance of low SBP according to DM in acute HF. *Methods.* We prospectively recruited 589 patients admitted with acute HF. DM was defined according to the 2011 American Diabetes Association recommendations. Patients were followed for 6 months and HF-death was the endpoint. A multivariate Cox-regression model was used to assess the prognostic impact of SBP. A stratified analysis according to DM was performed. *Results.* Median patients' age was 79 years and DM was present in 50.8%. Ischemic aetiology HF and hypertension history were more common in diabetics. Diabetic patients had worse renal function and lower total cholesterol and were more often discharged with antiplatelet therapy and statin. During followup, 89 patients died due to HF. The multivariate-adjusted HR for the 6-month HF death in non-diabetic patients with an admission SBP < 115 mmHg (1st quartile) was 2.94 (95% CI: 1.49–5.79), while lower admission SBP was not associated with HF mortality in diabetics. *Conclusions.* The blood pressure paradox in HF is only observed in non-diabetic HF patients. Diabetic patients seem to be a particular subgroup of HF patients.

## 1. Introduction

Hypertension and diabetes mellitus (DM) are well known risk factors for cardiovascular disease and mortality [1, 2].

Patients with DM are at increased risk of developing heart failure (HF) [3] and DM is a frequently encountered comorbidity in patients hospitalized with acute HF with reported prevalence ranging from 24.5% to 45% [4–9]. Patients with both conditions appear to have an increased morbidity and mortality risk, particularly those with ischemic HF [8–12]. 

Hypertension is also associated with increased risk of developing HF [13]. However, once HF is established, patients with higher systolic blood pressure (SBP) appear to have better prognosis. This survival benefit is present in both chronic stable HF with or without left ventricular systolic dysfunction (LVSD) [14–17] as well as in acute decompensated HF irrespective of left ventricular function [4–6, 11, 17–23]. This well recognized and increasingly accepted paradox is still not completely understood. It is not apparent if low SBP has a true casual relation with worse outcome or if it merely represents a bystander [24]. 

When treating hypertension, the blood pressure goal in diabetic patients is more ambitious than that in non-diabetics. In diabetic patients, the lower the better rule is well accepted [2]. However, if HF is also a comorbidity, the problem may be more difficult to solve in everyday practice.

Although a recent study [7] in acute HF patients with DM found that SBP < 100 mmHg was independently related to in-hospital mortality, a direct comparison of this paradoxical behavior of SBP between diabetic and non-diabetic patients in order to study if this reverse epidemiology phenomenon applies equally no matter the DM history was never specifically addressed.

We aimed to study if the 6-month HF mortality predicted by low admission SBP in patients hospitalized due to acute HF was different according to DM history.

## 2. Methods

Between March 2009 and December 2010, all patients presenting to the emergency department of our tertiary care academic hospital with dyspnoea and/or other HF symptoms and admitted in the internal medicine ward with the primary diagnosis of HF were eligible for inclusion in an acute HF prospective observational registry. Both patients with worsening or *de novo* HF entered the study. Exclusion criteria were: (1) patients with acute coronary syndromes; (2) patients with no echocardiographic structural or functional cardiac abnormalities; and (3) patients whose complaints were attributed by the attending physician to causes other than HF. 

Treatment decisions, timing of discharge, and discharge medication were at discretion of the attending physician and the physicians, were aware of the ongoing registry. 

An echocardiogram was performed within 72 h of admission. Comprehensive echocardiographic assessment was performed using a multifrequency matrix probe (Vivid6, GE Healthcare, Chalfont St Giles). HF diagnosis was made according to the European Society of Cardiology guidelines [25]. Both patients with LVSD and those with HF with preserved ejection fraction were studied. As a general reference, severe LVSD corresponded to left ventricular ejection fraction (LVEF) lower than 30%, moderate LVSD to LVEF between 30 and 40%, and mild to LVEF between 40 and 50%. LVEF above 50% was assumed as normal systolic function.

A fasting venous blood sample was collected from all patients between 8 and 9 am on the discharge day. Plasma B-type natriuretic peptide (BNP) was measured by way of a chemiluminescent immunoassays using an Architect i 2000 automated analyzer (Abbott, Lisboa, Portugal). Serum sodium, creatinine, urea, and total cholesterol were measured using conventional methods with an Olympus AU5400 automated clinical chemistry analyzer (Beckman-Coulter, Izasa, Porto, Portugal). Haemoglobin was obtained using an automated blood counter Sysmex XE-5000 (Emilio de Azevedo Campos, Porto, Portugal). Serum high-sensitivity C-reactive protein (hsCRP) was assayed using particle-enhanced immunonephelometric assays on a BN II laser nephelometer (Siemens, Lisboa, Portugal). Glycosylated haemoglobin (HbA1c) was determined by a ion-exchange high-performance liquid chromatography system with a D-10 Bio-Rad analyzer (Bio- Rad, Porto, Portugal).

Admission clinical data, demographic data, and comorbidities as well as discharge medication were recorded.

Arterial hypertension was defined as the presence of previous diagnosis, record of antihypertensive pharmacological treatment, or blood pressure above 140/90 mmHg. Coronary heart disease (CHD) was defined as history of acute myocardial infarction or significant CHD image confirmed. Anaemia was considered when haemoglobin level was below 13 g/dL in men or 12 g/dL in women. Hyponatremia was considered when serum sodium was below 135 mEq/L. Renal dysfunction was considered when plasma creatinine exceeded 1.5 mg/dL.

DM was defined as either a known previous diagnosis, current prescription of either an oral hypoglycemic agent or insulin, a fasting venous blood glucose above 126 mg/dL, or a random glucose >200 mg/dL. In addition, all patients with a glycosylated haemoglobin above 6.5% were also considered diabetic [2].

The endpoint under study was HF death. Patients were followed for a 6-month period after hospital discharge. Follow-up was made by consulting hospital registries of each patient and by telephone contact. One patient was lost during follow-up.

The study protocol conforms to the ethical guidelines of the declaration of Helsinki and was approved by the local ethics committee.

### 2.1. Statistical Analysis

Continuous variables are presented as mean (standard deviation) or median (interquartile range) if the distribution is skewed; categorical variables are presented as counts and proportions. Diabetic patients were compared with non-diabetic ones. A*χ* ^2^ test was used for categorical variables, an independent samples *t*-test for normally distributed continuous variables, and the Mann-Whitney *U* test was used when the distribution was not normal. 

We used a Cox-regression analysis to quantify the prognostic impact of admission SBP. The effect of SBP was assessed according to two categories using the cut-off of 115 mmHg. The cut-off used corresponded to the 25th percentile and was the cut-off used in the ADHERE registry [5].

The difference in the effect of SBP according to DM was formally tested by inclusion of an interaction term between SBP (115 mmHg used as cut-off for categorization) and DM.

We built a multivariate Cox-regression model to analyze the prognostic impact of SBP. Variables with prognostic impact in a univariate approach in either of the groups and variables classically known to influence HF prognosis were included in the final model. After documentation of a significant interaction, separate models were fitted in the two subgroups of patients according to DM history, to quantify the specific effect of SBP in each group.

We stored and analysed data using SPSS (SPSS Inc., Chicago, Illinois, 20.0).

## 3. Results

During the study period, a total of 589 patients were discharged after an admission due to acute HF. Medium length-of-stay was 8 days (interquartile range: 6–11); median patients' age was 79 years (interquartile range: 72–84) and 44.3% of the patients were men. HF aetiology was ischemic in 235 patients (39.9%); 76.7% of the patients had history of hypertension and 55.3% of the patients had systolic HF. DM was a comorbidity in 50.8% of the patients as defined by the recent American Diabetes Association (ADA) recommendations. Patients' characteristics and the comparison between diabetic and non-diabetic patients are shown in Table 1. In diabetic patients, HF was more often of ischemic aetiology; hypertension was also more common in diabetics. Diabetic patients had worse renal function, lower total cholesterol, higher body mass index and were more often medicated with statin therapy and antiplatelet drugs. Patients were not different in clinical characteristics at presentation, nor in discharge laboratory parameters. Importantly, admission SBP was similar in both groups.

Upon admission, diabetic patients were more often on medications with hypotensive effect: angiotensin-converting enzyme inhibitors (ACEi) and/or angiotensin receptor blockers (ARB), calcium channel antagonists, nitrates, and diuretics, than their non-diabetic counterparts (data not shown). 

One hundred and forty-six (24.8%) patients had an admission SBP < 115 mmHg: 75 (25.9%) non-diabetics and 71 (23.7%) diabetics, *p* value 0.55.

**Figure 1:**
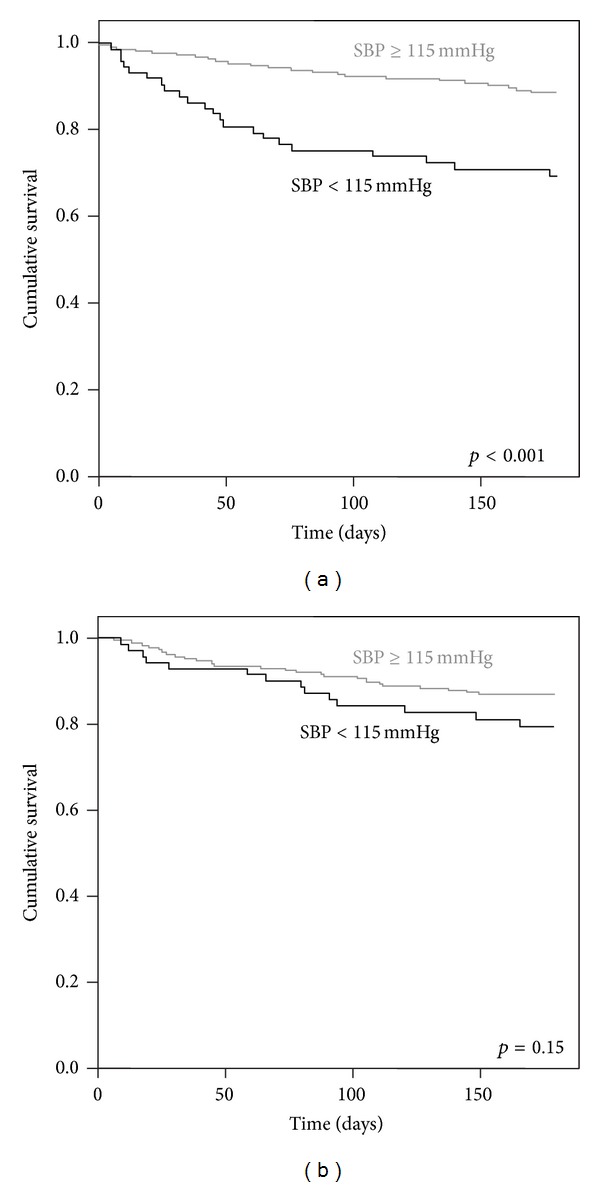
Kaplan-Meier survival curves according to admission SBP using 115 mmHg as cut-off, in non-diabetic and diabetic patients. Non-diabetic patients with an admission SBP < 115 mmHg had higher 6-month risk of HF death than those with admission SBP ≥ 115 mmHg (a). In diabetic patients, no difference in HF death risk existed between those admitted with SBP below 115 mmHg or above.

During the 6-month follow-up, 89 patients died due to HF: 45 non-diabetic and 44 diabetic patients. Kaplan-Meier estimates showed a significantly higher risk of death for non-diabetic patients with an admission SBP below 115 mmHg and no difference according to these SBP categories in diabetic HF patients. Figure 1 shows the Kaplan-Meier 6-month survival curves according to admission SBP (115 mmHg used as the cut-off value) in both diabetic and non-diabetic HF patients.

Table 2 shows predictors of 6-month HF mortality according to DM history. Predictors of 6-month HF death in non-diabetic patients were atrial fibrillation, lower admission SBP, anaemia, higher BNP at discharge, and prescription with ACEi and/or ARB. Predictors of 6-month HF death in diabetic patients were atrial fibrillation, hypertension history, total cholesterol <  125 mg/dL, higher discharge BNP, and prescription with prognostic modifying therapy.

In the whole group of 589 patients, an admission SBP < 115 mmHg (1st quartile) is associated with 6-month HF death with an HR of 2.27 (95% CI 1.49–3.47); however, this association of lower pressure with shorter survival was only reproduced in the subgroup of non-diabetic patients with an HR of 3.16 (95% CI: 1.76–5.67). No such association was observed in the diabetic HF patients.

The association of SBP with 6-month HF death in non-diabetic HF patients was independent of the other prognostic factors.  Table 3 shows the final multivariate models, separately for diabetic and non-diabetic patients. The multivariate-adjusted HR of 6-month HF death in non-diabetic acute HF patients with an admission SBP < 115 mmHg was 2.94 (95% CI: 1.49–5.79), while a lower admission SBP was not associated with HF mortality in the subgroup of diabetic patients, *p* for interaction = 0.04.

## 4. Discussion

We report that, in a group of acute HF patients, low SBP at admission is associated with adverse 6-month prognosis only in non-diabetic patients. Using the recent ADA recommendation, the prevalence of DM is higher than previously reported. 

The multivariate-adjusted risk of 6-month HF death among the non-diabetic patients with an admission SBP below 115 mmHg was almost 3-fold higher than for those with higher admission SBP. There was no association between lower SBP at admission and worse prognosis in the subgroup of diabetic patients. The difference of the admission SBP prognostic impact according to DM was statistically significant (*p* for interaction between SBP and DM = 0.04). 

Several studies have reported that lower SBP predicts worse outcome both in the acute HF setting [4–6, 11, 17–23] and in a chronic HF context [14–17]. The reason for this blood pressure paradox, like other reverse epidemiology phenomena in HF, is not completely understood. Low SBP may simply mirror poorer myocardial reserve and have no causal effect in HF survival, behaving as a marker of disease severity [14, 17–20, 24]. Contrarily, decreased blood pressure and end-organ hypoperfusion with renal dysfunction, cognitive impairment, and muscle wasting have been proposed to contribute to increased mortality [18–21]. This problem may rise more intensely to the clinician when managing an acute HF patient, in which case therapeutic optimization, whether with symptomatic therapy or prognostic modifying therapy, usually courses with blood pressure lowering. Physicians may feel reluctant to start or increase doses of drugs that are well known to relieve congestive symptoms and improve HF survival, because of their hypotensive effect, in this particular group of patients presenting with low SBP [19, 26]. 

HF is a condition of paradoxes; however, the universal applicability of such paradoxes to the group of HF patients as a whole has been questioned [27]. Our results in the non-diabetic group of HF patients were consistent with the well-known blood pressure paradox. However, in the diabetic patients this paradox was not observed. In our population of acute HF patients, low SBP predicted shorter survival only in non-diabetics. 

To the best of our knowledge, this is the first study in acute HF patients that analyses and compares the impact of SBP separately in diabetic and non-diabetic patients. The significant difference in low SBP prognostic impact between diabetic and non-diabetic patients is intriguing and challenging and clinical implications are difficult to predict. It is possible that in diabetic HF patients a lower SBP is more likely attributable to cardiovascular autonomic dysfunction (CAD) and less likely a mirror of a “pump failure” process. According to this hypothesis, a lower blood pressure would not be a marker of HF severity but instead represents a DM complication. CAD is a frequent complication of DM and may be present early in the course of the disease [28]. Diabetic patients with CAD are prone to present orthostatic hypotension, resting tachycardia, exercise intolerance, and cardiovascular lability [28]. This could explain why in a stress situation, such as an acute HF episode, diabetic patients can present with lower blood pressure without necessarly associated worse outcome. 

If this is true, it would favor the hypothesis that, in non-diabetics, a lower blood pressure is, in fact, a marker of disease severity and not associate *per se* to shorter survival. In diabetics, a lower SBP does not necessarily mean more severe HF but possibly CAD and therefore does not behave as an innocent bystander in an association with mortality. This suggests that physicians should not be deterred by admission blood pressure to optimize HF drug therapy, provided that end-organ perfusion is assured. This is particularly valid for diabetic patients.

In acute HF studies and registries, diabetic patients have been reported to be more frequently prescribed with blood pressure lowering drugs upon admission than non-diabetics [7–9]. The same occurred with our diabetic group of HF patients. To some extent, SBP in the lower range at admission in the diabetic group may have reflected the heavier use of such drugs and not a worse cardiac function.

The blood pressure paradox, as others, appears not to be applicable to all HF patients. Diabetic HF patients seem to be a very specific group of HF patients in which paradoxes tend to not be observed [27].

In our population of 589 patients that were admitted due to acute HF (worsening or *de novo*), the prevalence of DM, defined according to recent ADA recommendations [2], is 50.8%, one of the highest reported. This is, as far as extensive literature review could retrieve, the first acute HF study that uses the most recent ADA recommendations for the diagnosis of DM. Despite the application of the most recent criteria and the higher prevalence of DM in our study, the characteristics of HF patients with DM were not different from those reported in other series [7–9].

This study has limitations. As a single center study in a tertiary care academic hospital, patients may not be representative of the entire HF population. Also, the relatively short number of events in analysis precludes firm conclusions. As the physicians were aware of the ongoing registry, a treatment bias cannot be excluded. Regardless of its prospective design, the establishment of causality relations is not possible and the blood pressure paradox remains to be comprehended in its essence. Despite all these limitations, this is a study on real world HF patients, old aged and with multiple morbidity, that seek medical help due to acute HF and treated according to what is considered the state of the art.

## 5. Conclusions

The blood pressure paradox in HF is only applicable to HF patients with no concomitant DM.

Diabetic patients should probably be looked at as a particular subgroup of HF patients with many yet to be defined specificities in care. 

## Figures and Tables

**Table 1:** Patients' demographic, clinical and laboratory characteristics, comparison between diabetic and non-diabetic patients.

	All patients (*n* = 589)	Non-diabetic (*n* = 290)	Diabetic (*n* = 299)	*p* value
Clinical characteristics				
Age (years), median (IQR)	79 (72–84)	80 (69–86)	78 (72–83)	0.06
Male sex, *n* (%)	261 (44.3)	131 (45.2)	130 (43.5)	0.68
Ischemic aetiology of HF, *n* (%)	235 (39.9)	100 (34.5)	135 (45.2)	0.008
Arterial hypertension history, *n* (%)	438 (76.7)	185 (66.1)	253 (86.9)	<0.001
Atrial fibrillation history, *n* (%)	269 (46.1)	134 (47.0)	135 (45.3)	0.68
LVSF, *n* (%)				
Preserved	255 (44.7)	131 (46.1)	124 (43.2)	
Mild LVSD	35 (6.1)	18 (6.3)	17 (5.9)	
Moderate LVSD	85 (14.9)	38 (13.4)	47 (16.4)	
Severe LVSD	196 (34.3)	97 (34.2)	99 (34.5)	0.76
NYHA class at admission (IV versus others), *n* (%)	355 (60.4)	163 (56.4)	192 (64.2)	0.05
SBP at admission (mmHg), median (IQR)	131 (115–152)	132 (113–151)	131 (115–152)	0.76
BMI at admission (Kg/m^2^), median (IQR)	25.2 (22.6–27.7)	24.3 (22.1–27.0)	25.9 (23.2–28.5)	<0.001
Laboratory at discharge				
Haemoglobin (g/dL), mean (SD)	11.8 (10.5–13.2)	12.0 (10.6–13.5)	11.8 (10.4–13.0)	0.20
Anaemia, *n* (%)	350 (60.2)	165 (57.9)	185 (62.5)	0.26
Creatinine (mg/dL), median (IQR)	1.30 (1.05–1.71)	1.28 (1.00–1.60)	1.37 (1.10–1.85)	<0.001
Renal dysfunction, *n* (%)	209 (35.8)	86 (30.1)	123 (41.4)	0.004
Sodium (mEq/L), median, IQR	138 (135–140)	138 (135–140)	137 (135–140)	0.54
Hyponatremia, *n* (%)	141 (24.4)	69 (24.4)	72 (24.3)	0.99
Total cholesterol (mg/dL), median (IQR)	151 (125–180)	156 (132–187)	142 (121–174)	0.002
C-reactive protein (mg/L), median (IQR)	12.8 (6.1–26.8)	12.9 (6.1–28.6)	12.8 (6.0–25.4)	0.50
BNP (pg/mL), median (IQR)	743.8 (304.0–1385.4)	722.8 (262.9–1326.6)	767.0 (337.0–1476.2)	0.20
Glycosylated haemoglobin (%), median (IQR)	6.2 (5.7–6.9)	5.8 (5.5–6.0)	6.9 (6.5–7.8)	<0.001
Discharge medication				
Beta-blocker, *n* (%)	445 (75.9)	212 (73.4)	233 (78.5)	0.15
ACEi or ARB, *n* (%)	466 (79.4)	220 (76.1)	249 (82.6)	0.05
Spironolactone, *n* (%)	137 (23.4)	77 (26.6)	60 (20.2)	0.08
Statin, *n* (%)	353 (61.6)	152 (54.1)	201 (68.8)	<0.001
Antiplatelet drugs, *n* (%)	382 (66.4)	166 (58.9)	216 (73.7)	<0.001

HF death, *n* (%)	89 (15.1)	45 (15.6)	44 (14.7)	0.77

ACEi: angiotensin converting enzyme inhibitor; ARB: angiotensin II receptor 1 blocker, BMI: body mass index; BNP: B-type natriuretic peptide; HF: heart failure; IQR: interquartile range; LVSD: left ventricular systolic dysfunction; LVSF: Left ventricular systolic function; NYHA: New York Heart Association; SBP: systolic blood pressure.

**Table 2:** Univariate association between patients characteristics and 6-month HF death in non-diabetic and diabetic decompensated HF patients.

	Non-diabetics HF death (*n* = 45) HR (95% CI)	*p* value	Diabetics HF death (*n* = 44) HR (95% CI)	*p* value
Clinical characteristics				
Age (per year)	1.02 (1.00–1.05)	0.08	1.02 (0.98–1.05)	0.36
Male sex	1.38 (0.77–2.48)	0.28	1.36 (0.75–2.45)	0.31
Ischemic aetiology of HF	1.04 (0.56–1.91)	0.90	1.10 (0.61–1.98)	0.76
Arterial hypertension history	0.62 (0.34–1.13)	0.12	0.47 (0.23–0.96)	0.04
Atrial fibrillation history	2.10 (1.14–3.89)	0.02	2.03 (1.11–3.72)	0.02
LVSD versus HFpEF	1.58 (0.86–2.91)	0.14	1.25 (0.68–2.33)	0.47
NYHA class at admission (IV versus others)	1.19 (0.68–2.17)	0.56	1.20 (0.64–2.26)	0.57
SBP at admission <115 mmHg	3.16 (1.76–5.67)	<0.001	1.58 (0.84–2.98)	0.16
BMI at admission (per Kg/m^2^)	0.94 (0.87–1.01)	0.08	0.97 (0.90–1.05)	0.45
Laboratory at discharge				
Anaemia	2.37 (1.20–4.69)	0.01	1.81 (0.91–3.58)	0.09
Renal dysfunction	1.39 (0.75–2.56)	0.30	1.54 (0.85–2.80)	0.16
Hyponatremia	1.17 (0.60–2.27)	0.65	1.76 (0.94–3.29)	0.08
Total cholesterol <125 mg/dL	1.79 (0.88–3.62)	0.10	1.98 (1.04–3.80)	0.04
C-reactive protein (per mg/L)	1.00 (0.99–1.01)	0.90	1.00 (0.99–1.01)	0.80
BNP (per 100 pg/mL)	1.02 (1.01–1.03)	<0.001	1.02 (1.01–1.03)	<0.001
Glycosylated haemoglobin (per %)	0.74 (0.36–1.52)	0.42	1.04 (0.85–1.26)	0.72
Discharge medication				
Beta-blocker	0.85 (0.44–1.61)	0.61	0.41 (0.22–0.76)	0.004
ACEi or ARB	0.35 (0.20–0.64)	0.001	0.41 (0.21–0.78)	0.002
Spironolactone	0.75 (0.37–1.51)	0.42	0.86 (0.40–1.85)	0.70
Statin	0.62 (0.34–1.13)	0.12	0.98 (0.51–1.88)	0.94
Antiplatelet drugs	1.22 (0.66–2.26)	0.52	0.98 (0.49–1.94)	0.95

ACEi: angiotensin converting enzyme inhibitor; ARB: angiotensin II receptor 1 blocker, BMI: body mass index; BNP: B-type natriuretic peptide; CI: confidence interval; HF: heart failure; HFpEF: Heart failure with preserved ejection fraction; HR: hazard ratio; LVSD: left ventricular systolic dysfunction; NYHA: New York Heart Association; SBP: systolic blood pressure.

**Table 3:** Final multivariate Cox-regression model for the effect of SBP below 115 mmHg on 6-month HF death after an acute HF episode, according to DM.

	Non-diabetics HR (95% CI)	*p* value	Diabetics HR (95% CI)	*p* value
SBP < 115 mmHg	2.94 (1.49–5.79)	0.002	1.11 (0.52–2.37)	0.80
Arterial hypertension history	0.71 (0.35–1.43)	0.34	0.53 (0.24–1.17)	0.12
Atrial fibrillation history	2.74 (1.32–5.69)	0.007	1.50 (0.74–3.04)	0.26
BNP (per 100 pg/mL)	1.02 (1.01–1.03)	0.001	1.02 (1.01–1.03)	0.001
Anaemia	1.93 (0.87–4.31)	0.11	2.04 (0.89–4.67)	0.09
Total cholesterol <125 mg/dL	1.22 (0.57–2.59)	0.61	1.51 (0.76–3.01)	0.24
Beta-blocker	0.86 (0.42–1.78)	0.69	0.39 (0.20–0.78)	0.007
ACEi and/or ARB	0.52 (0.26–1.06)	0.07	0.40 (0.20–0.81)	0.01

ACEi: angiotensin converting enzyme inhibitor; ARB: angiotensin II receptor 1 blocker; BNP: B-type natriuretic peptide; CI: confidence interval; HR: hazard ratio; SBP: systolic blood pressure.
